# Performance of five free large language models in dental trauma: a 30-day longitudinal benchmark study

**DOI:** 10.3389/froh.2025.1737114

**Published:** 2025-12-15

**Authors:** Rafaela Mancini Lisboa, Arian Braido, Adriana de-Jesus-Soares, Nitesh Tewari, Carlos José Soares, Luiz Renato Paranhos, Walbert A. Vieira

**Affiliations:** 1 Departament of Dentistry, Centro Universitário das Faculdades Associadas de Ensino – UNIFAE, São João da Boa Vista, Brazil; 2 Division of Endodontics, Department of Restorative Dentistry, Piracicaba Dental School, Universidade Estadual de Campinas - UNICAMP, Piracicaba, Brazil; 3 Division of Pediatric and Preventive Dentistry, Centre for Dental Education and Research, All India Institute of Medical Sciences, Delhi, India; 4 Department of Operative Dentistry and Dental Materials, School of Dentistry, Universidade Federal de Uberlândia, Uberlândia, Brazil; 5 Department of Orthodontics, Universidade Federal de Uberlândia, Uberlândia, Brazil

**Keywords:** artificial intelligence, chatbot, dental trauma, large language models, traumatic dental injuries

## Abstract

**Objective:**

To compare the accuracy and consistency of five large language models (LLMs) in generating responses about dental trauma.

**Materials and methods:**

Sixty dichotomous (true/false) questions were submitted daily to each LLM (ChatGPT, Google Gemini, Microsoft Copilot, DeepSeek, and Meta AI) for 30 days, totaling 18,000 responses. All interactions were performed under two prompting conditions (zero-shot and zero-shot with context). LLM responses were compared against the International Association of Dental Traumatology (IADT) guidelines. Statistical analysis was conducted using a generalized linear mixed model (GLMM) with a binomial distribution (*α* = 0.05), alongside calculation of sensitivity, specificity, accuracy, and area under the ROC curve (AUC) based on the 60-item set. Temporal stability was assessed using the intraclass correlation coefficient ICC.

**Results:**

All LLMs achieved accuracy above 85%, with Microsoft Copilot (91.1%) and DeepSeek (90%) performing best; no significant difference was observed between them (*p* > 0.05), but both outperformed the other models (*p* < 0.05). DeepSeek and Microsoft Copilot also showed the highest consistency over 30 days (ICC > 0.90).

**Conclusion:**

All evaluated LLMs, particularly Copilot and DeepSeek, demonstrated high accuracy in providing information on dental trauma, with stable performance over time. While the use of a context prompt did not significantly affect accuracy or stability.

## 1 Introduction

Large language models (LLMs) are AI systems that generate human-like responses using natural language processing and deep neural networks (1). Unlike search engines, they deliver information conversationally, aiding understanding of complex topics (2).

The popularization of LLMs began in late 2022 with the public release of ChatGPT by OpenAI (3). Since then, their use as accessible sources of medical guidance has been considered promising (4–7). However, limitations such as hallucinations (incorrect but convincing answers) and bias in training data remain significant challenges (8).

Dental trauma accounts for a considerable proportion of dental emergencies (9, 10) and is recognized as a global public health problem (11, 12). Its severity varies, causing pain, functional, phonetic, and esthetic impairment, and may ultimately lead to tooth loss (13, 14). Immediate management is critical for prognosis (15, 16), yet misinformation among both professionals and laypeople remains a barrier (17–19). Limited clinical exposure during undergraduate training, combined with controversial and weakly evidence-based recommendations, further increases the risk of mismanagement (20, 21).

To address these issues, treatment guidelines and professional orientation platforms have been developed to disseminate knowledge and standardize management worldwide (22–25). Given the potential of LLMs as tools for health information dissemination, several studies have already evaluated their accuracy in the context of dental trauma (26) and general dentistry (27–31). In past 2 years, several research groups (32–36) have tested the adequacy of responses of different LLMs with variability in the number and types of questions asked, the LLM models tested, and the outcome variables assessed. One study assessed Gemini using questions derived from the European Society of Endodontology (ESE) guidelines, reporting an accuracy of 80.8% (35). Another compared six chatbots—ChatGPT 3.5, ChatGPT 4.0, Gemini, Copilot, Perplexity, and ChatGPT 4.0 Plus—across 972 interactions with 18 questions on tooth avulsion (33). ChatGPT 4.0 Plus achieved the highest accuracy (95.6%), whereas Perplexity showed the lowest (67.2%) (33). Despite their relevance, these studies did not assess the longitudinal consistency of responses and excluded newer models such as ChatGPT-4o, DeepSeek 3.0, and MetaAI (33, 35).

With the rapid evolution of LLM algorithms and architectures, continuous updating of these data is essential, incorporating more recent and robust models. Therefore, the primary objective of this study was to compare the accuracy and longitudinal consistency (30 days) of five modern, freely accessible LLMs (Copilot, DeepSeek, ChatGPT-4o, Gemini, and MetaAI) as sources of information on dental trauma, using IADT guidelines as the gold standard. The secondary objective was to assess whether interaction strategies (zero-shot vs. few-shot learning) influence their performance.

## 2 Materials and methods

This observational longitudinal study did not involve human subjects or identifiable data. Ethics approval was therefore not required. Reporting followed TRIPOD-LLM guidelines (37) (Supplementary Material 1) and the CHART statement (38).

### 2.1 Benchmark development

Two endodontists (WAV and AB) with five years' dental trauma experience developed a 60-question true/false benchmark. Items covered diagnosis and management of traumatic dental injuries in primary and permanent teeth, based on the latest IADT guidelines (Supplementary Material 2). *Ground-truth* answers were set by consensus between the two dentists.

The IADT guidelines were selected as the primary reference because they represent expert consensus and evidence-based recommendations in dental trauma. Three external experts (two endodontists, one pediatric dentist; >5 years of experience) validated the benchmark questions for clarity and objectivity. Questions targeted key clinical decisions, differential diagnoses, complications, and emergency care. The dichotomous format minimized ambiguity and allowed objective accuracy measurement. The benchmark enabled both cross-sectional and longitudinal comparisons.

### 2.2 Evaluation protocols

For each interaction, new accounts were created for the five different LLMs included in this study, selected for their wide free availability and diverse architectures (Table 1). All models were accessed through the Google Chrome browser (no active history, cache, or logged-in chat history) to prevent any influence from prior interactions. All interactions were conducted in Portuguese to ensure linguistic consistency and to assess performance in a non-English clinical context. Testing took place between February and March 2025, and no frozen or version-locked API endpoints were available during data collection; responses therefore reflect the publicly deployed model versions at that time.

**Table 1:** Specifications of the LLMs used in this study.

Model	Platafor	Access type	Architecture
ChatGPT-4o	OpenAI (https://www.chat.openai.com)	Free/Proprietary	Transformer, Instruct-tuned
Gemini 2.5 Flash	Google (https://www.gemini.google.com)	Free/Proprietary	Transformer multimodal
Microsoft Copilot	Microsoft (https://www.copilot.microsoft.com)	Free/Integrated	GPT-4 based
DeepSeek 3.0	DeepSeek (https://www.deepseek.com)	Free/Open API	Chinese LLM (bilingual)
Meta AI (LLaMA 4)	Meta (https://www.meta.ai)	Free/Proprietary	LLaMA-based, fine-tuned

A single trained researcher (RML) generated 60 responses daily for 30 consecutive days, always in the late afternoon (4–5 p.m.), to minimize potential performance variability due to server load or update cycles. A fresh conversation window was initiated before each testing block, and session data were cleared to ensure that no memory from previous interactions was retained. Models were tested under two conditions:

Zero-shot: questions presented directly, without context (e.g., “An avulsed primary tooth should never be replanted. True or false?”).
Zero-shot with context: questions preceded by the instruction “Answer as a dentist, following the most recent IADT guidelines. An avulsed primary tooth should never be replanted. True or false?”

To fully isolate the two conditions, the contextual interactions were always conducted in a new chat, independent from the zero-shot session of the same day.

This approach simulated two usage scenarios: a lay user and a professional familiar with prompt engineering. It allowed us to observe the impact of calibration on model behavior (*prompt conditioning*). In total, 18,000 interactions were collected (60 questions × 5 models × 2 conditions × 30 days). Responses were stored in a purpose-built Excel spreadsheet (Microsoft, Redmond, USA) and compared with the reference key. The same researcher who generated the responses also tabulated the data. A second researcher, blinded to the chatbot and not involved in data collection, evaluated each response as “correct” or “incorrect” based on the predefined *ground truth*.

### 2.3 Statistical analysis

Data were analyzed in R (v4.5, R Core Team, Vienna, Austria). Sensitivity, specificity, and AUC were computed from the pooled binary responses obtained across all testing days. This aggregation was used to represent the overall diagnostic performance of each model. Accuracy and 95% confidence intervals (95% CI) were estimated using the 60 unique items as the unit of analysis. For each Model × Prompt cell, accuracy was first computed at the item level (proportion of correct responses for each of the 60 items across the 30 repeated interactions). The overall accuracy for each cell was then obtained as the means of these 60 item-level accuracies. Precision and 95% CI were quantified using the standard error of a proportion based on 60 items.

Chatbot accuracy was compared using a Generalized Linear Mixed Model (GLMM) with binomial distribution and logistic link. Fixed effects included *LLM* (five levels) and *Prompt* (two levels), as well as their interaction (*LLM* *×* *Prompt*), forming a 5 × 2 factorial design. Random effects included questions (to account for variation in topics) and day (to account for temporal variation). Differences were tested with Wald χ^2^ and likelihood ratio tests (LRT). *Post hoc* pairwise comparisons were conducted using Tukey-adjusted contrasts, and results were reported as odds ratios (OR) with 95% CIs. Effect sizes were computed using Cohen's h, appropriate for proportional data, and interpreted as small (0.20), medium (0.50), or large (0.80) according to Cohen (39). Finally, A *post hoc* power analysis was performed using the standard errors of the GLMM fixed effects to estimate the minimum effect detectable with 80% power at *α* = 0.05. For each fixed effect (*Model, Prompt, and Model* *×* *Prompt*), the minimum detectable log-odds difference was computed as 2.8 × SE and converted to odds ratios. These values were compared with the magnitude of observed effects to determine whether the study had sufficient power to detect realistic differences among LLMs.

Day-to-day agreement was evaluated using the intraclass correlation coefficient (ICC) treating items as targets and days as raters (two-way random effects, absolute agreement, single measures) and its 95% CI. ICC values ≥0.75 were interpreted as good reliability, 0.5–0.75 as moderate, and <0.5 as poor stability.

## 3 Results

**Figure 1:**
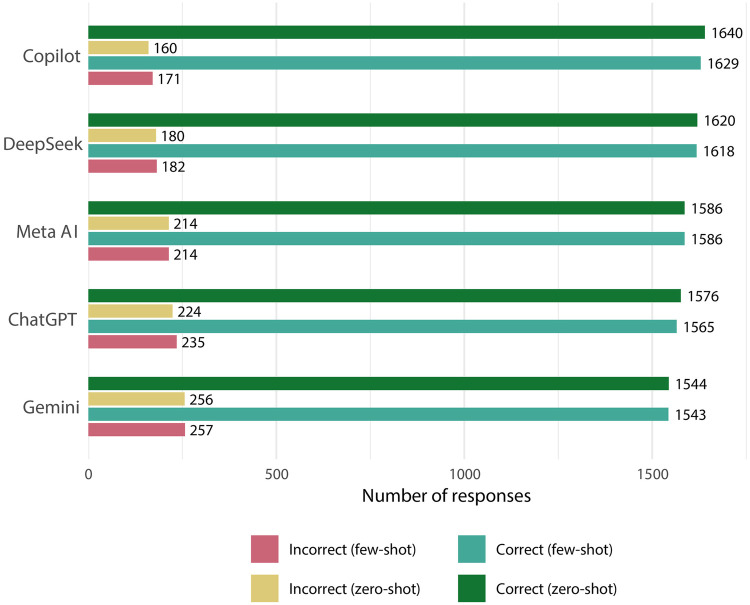
Proportion of correct and incorrect responses for each LLM under both prompting conditions.

**Figure 2:**
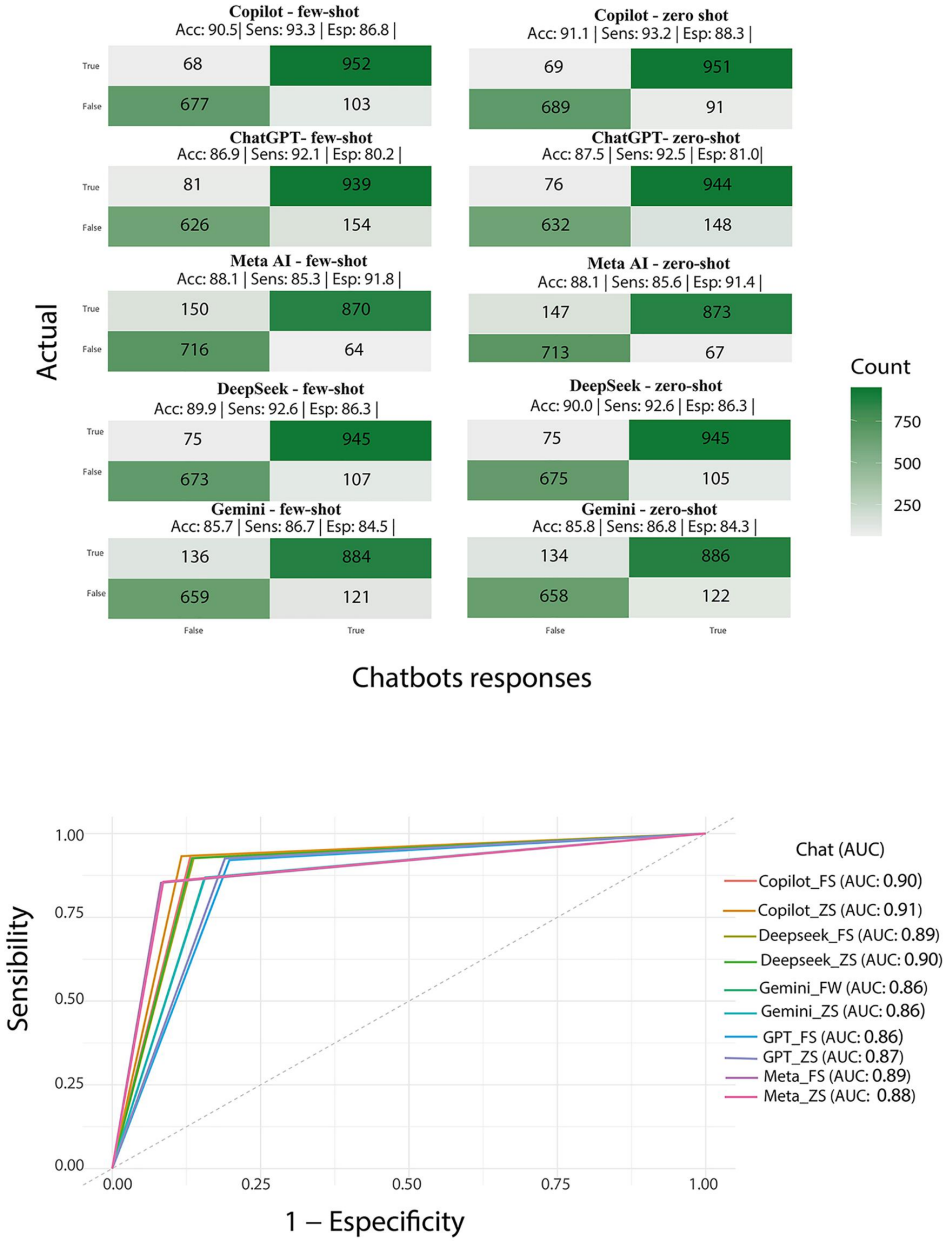
Confusion matrices, performance metrics, and ROC curve for each LLM. ACC, accuracy; Sens, sensitivity; Spec, specificity; CP, with prompt; SP, without prompt.

A total of 18,000 responses were generated, and none was excluded from analysis. Copilot showed the highest accuracy [zero-shot: 0.91 (95% CI: 0.84;0.98); with context: 0.91 (95% CI: 0.83;0.98)] followed by DeepSeek [zero-shot: 0.90 (95% CI: 0.82; 0.98); with context: 0.90 (95% CI: 0.82; 0.97)], MetaAI [zero-shot: 0.88 (95% CI: 0.80;0.96); with context: 0.88 (95% CI: 0.80;0.96)], ChatGPT (zero-shot: 0.88 (95% CI: 0.79; 0.96); with context: 0.87 (95% CI: 0.78; 0.95), and Gemini [zero-shot: 0.86 (95% CI: 0.77;0.95); with context: 0.86 (95% CI: 0.77;0.95)] ((Figure 1). Performance metrics are detailed in Figure 2. Copilot achieved the highest sensitivity (0.93) and AUC (0.90), while Meta AI showed the best specificity (0.92–0.91).

The factorial GLMM significantly improved model fit compared to the null model [χ^2^(9) = 148.6, *p* < 0.0001]. The main effect of LLMs was significant (χ^2^ = 75.66, *p* < 0.001), whereas Prompt (χ^2^ = 0.76, *p* = 0.38) and the LLMs × Prompt interaction (χ^2^ = 0.99, *p* = 0.91) were not. Random effect variances indicated substantial question-level heterogeneity (*σ*^2^_question = 23.12; SD = 4.81) and minimal day-to-day variability (*σ*^2^_day = 0.10; SD = 0.32).

Pairwise Tukey-adjusted comparisons revealed that Copilot and DeepSeek were superior to ChatGPT and Gemini (*p* < 0.01) (Table 2). Interactions with context showed similar results (Table 2). Effect size analysis indicated that differences between models were small (cohen's *d* range: 0.02–0.17). The *post hoc* power analysis indicated that the study had >80% power to detect main effects of *LLMs*, *Prompt*, and *Model* *×* *Prompt* interaction effects, confirming that the sample size was sufficient to detect effects of realistic magnitude (Supplementary Material 3).

**Table 2:** Pairwise comparisons between different types of chatbots with tukey adjustment and Cohen's h effect size.

Chatbot	Zero-shot			Zero-shot with context		
	OR (95% CI)	*p*-value	Cohen's h	OR (95% CI)	*p*-value	Cohen's h
Reference: ChatGPT						
Copilot	2.64 (1.51; 4.62)	<.01	0.115	2.50 (1.45; 4.30)	<.01	0.113
DeepSeek	1.88 (1.09; 3.22)	<.01	0.077	2.09 (1.23; 3.55)	<.01	0.092
Gemini	0.68 (0.41; 1.11)	0.28	0.052	0.77 (0.47;1.25)	0.80	0.035
MetaAI	1.14 (0.68; 1.90)	1.00	0.016	1.31 (0.79; 2.18)	0.81	0.035
Reference: Copilot						
DeepSeek	0.71 (0.40; 1.28)	0.71	0.038	0.83 (0.47; 1.48)	0.99	0.021
Gemini	0.26 (0.15; 0.45)	<.01	0.167	0.31 (0.18; 0.53)	<.01	0.149
MetaAI	0.43 (0.25; 0.76)	<.01	0.099	0.53 (0.30; 0.91)	<.01	0.077
Reference: DeepSeek						
Gemini	0.36 (0.21; 0.61)	<.01	0.130	0.37 (0.22; 0.62)	<.01	0.128
MetaAI	0.61 (0.35; 1.05)	0.11	0.061	0.62 (0.36; 1.08)	0.17	0.057
Reference: Gemini						
MetaAI	1.68 (1.02; 2.77)	0.03	0.069	1.70 (1.03; 2.80)	0.03	0.071

OR, odds ratio; CI, confidence interval.

**Figure 3:**
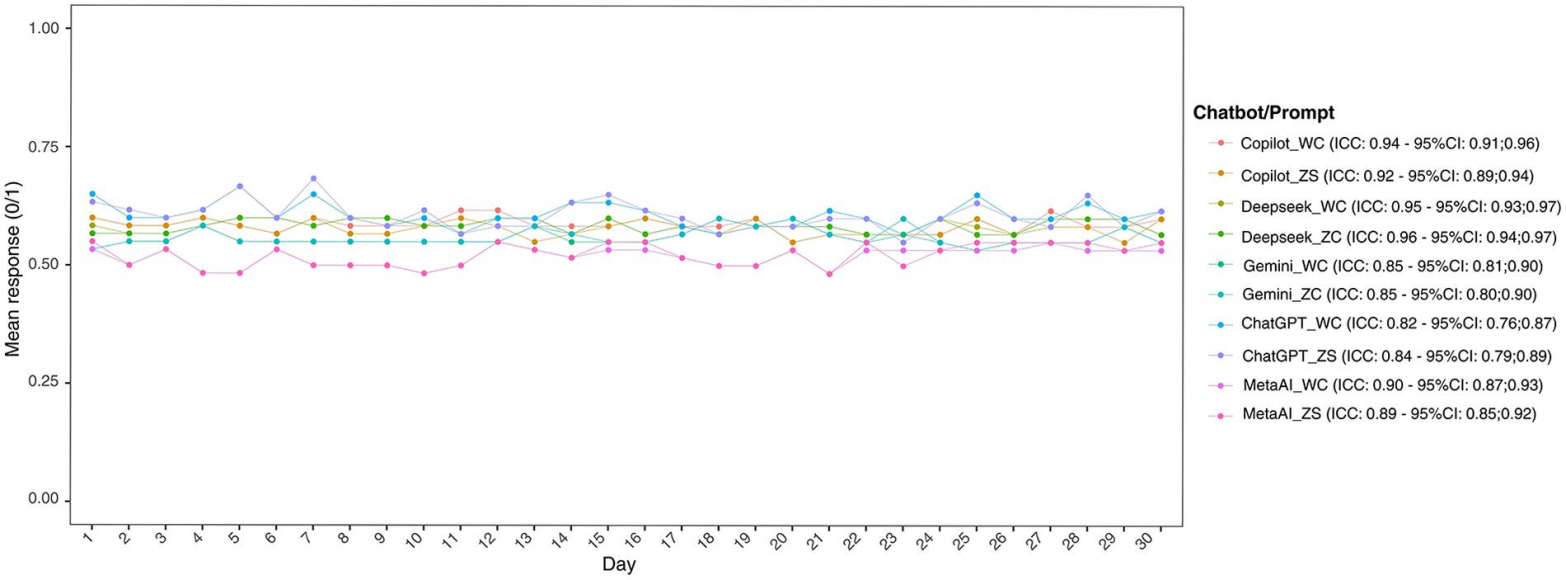
Mean responses (0 – false; 1 – true) of each LLM with zero-shot or with context prompt. Day-to-day agreement was evaluated using the intraclass correlation coefficient (ICC). ZS, zero shot; WC, with context.

All models showed good consistency over the 30 days, with Deepseek (zero-shot) (ICC: 0.95; 95% CI: 0.94–0.97) and Copilot (with context) (ICC: 0.94; 95% CI: 0.91; 0.96) standing out (Figure 3). The use of the prompt did not significantly alter the consistency of any LLM.

## 4 Discussion

This study evaluated the performance of five AI-based chatbots as information sources for dental trauma decision-making, using two interaction modes. All models showed high accuracy, with Microsoft Copilot and DeepSeek performing best. The use of context prompts did not significantly affect accuracy.

All models achieved accuracy above 85%, indicating that current LLMs are reliable sources for dental trauma information. Previous studies reported wider variability, with accuracy starting from 40% (32–34, 40, 41). Our results suggest an upward trend in chatbot accuracy compared to earlier versions (33, 35, 36), likely due to continuous model updates (41). In comparison with other fields of dentistry, our study showed accuracy levels consistent with those reported in prior research (30, 31).

That study showed that Microsoft Copilot achieved the best performance across multiple evaluation metrics. This result aligns with previous research (30), which demonstrated Copilot's superiority as an information source in answering multiple choice questions in dentistry. Another study reported higher accuracy (94.4%) for Microsoft Copilot in resolving complex osteoarticular infection cases compared with ChatGPT-4o (85.7%) and Gemini 2.0 Flash (86.5%) (42). In the context of dental trauma, our results are consistent with Mustuloğlu et al. (33), where Microsoft Copilot (80.8%) outperformed the free version of ChatGPT-4 (79.6%) and Google Gemini (78.3%). However, in the same study, Copilot performed worse than the paid version of ChatGPT-4o (95.6%). This performance may be explained by recent technical updates to Copilot, including access to medical information sources and refined embeddings for healthcare terminology (42).

Our study also found that DeepSeek achieved significantly better results than ChatGPT-4o and Gemini 2.5 Flash. DeepSeek uses a transformer architecture, is trained on a specialized multilingual corpus, and incorporates information sources in English and Chinese, expanding its training for clinical scenarios (128k tokens) (43). DeepSeek-R1 has shown higher accuracy (86.2%) in complex ophthalmology cases compared with Gemini 2.0 Pro (71.5%) and ChatGPT (69.2%) (44). In dentistry, ChatGPT and DeepSeek have demonstrated superior accuracy compared with Gemini for multiple-choice questions on fixed prosthodontics (45). This is the first study to assess DeepSeek for dental trauma. Further research should use varied Q&A methods to verify its effectiveness and review response quality and readability (46).

In this study, specificity was calculated to evaluate the models' ability to identify false alternatives. This metric is important because widely used chatbots are trained on internet data, making them susceptible to disseminating incorrect information (46, 47). All models achieved specificity above 80%, with Meta AI reaching 91.8%. These findings suggest that chatbots can effectively identify and classify false information, showing promise in reducing the spread of misinformation on clinical practices in dental trauma. Previous research has highlighted the relatively low accuracy of Llama-based chatbots compared to ChatGPT when addressing multiple-choice questions in prosthodontic and restorative dentistry (28). These findings emphasize the variability in performance among different LLMs depending on the dental specialty and question format. Notably, this study is the first to apply dichotomous question evaluation metrics to LLMs in the context of dental trauma. By employing this approach, the study provides a new perspective on chatbot performance within this specific clinical scenario. Further research is recommended to validate these results and extend the assessment to other dental specialties, ensuring comprehensive understanding of LLM effectiveness across various contexts in dentistry.

A key contribution of this study was the longitudinal analysis of response consistency over 30 consecutive days. Models such as Microsoft Copilot and DeepSeek showed high stability throughout the period, consistent with previous reports (32, 33, 35, 41). This outcome is crucial as it indicates that these models provide consistent information in routine clinical situations, ensuring reliability in dental trauma guidance.

Although calibration prompts improved consistency for some LLMs, they did not significantly enhance accuracy for any chatbot, contrary to findings in other medical studies (48, 49). This result may be due to technical factors, such as variable contextualization capacity or training data bias, or methodological factors, such as insufficient prompt specificity or model adaptation (50). Future research should investigate optimized and adaptive prompt engineering for each LLM architecture to improve accuracy in complex clinical contexts, including dental trauma (51, 52).

This study has limitations. First, we evaluated only free, web-accessible chatbot versions, so future studies should include a wider range of LLMs to enhance generalizability. The dichotomous question-and-answer format may not capture the full complexity of clinical reasoning, especially when multiple management options exist (30, 31), and may inflate apparent accuracy. A recent systematic review with network meta-analysis highlighted substantial variability in LLM accuracy depending on question type: ChatGPT-4o (SUCRA = 0.9207) demonstrated strong performance in terms of accuracy for objective questions, while ChatGPT-4 (SUCRA = 0.8708) and followed by Claude 2.1 (SUCRA = 0.7796) excelled at answering open-ended questions (41). Therefore, future studies should include multiple-choice questions and simulated clinical cases (image and text) to broaden applicability (30, 31, 55) specially in considering dental trauma context (32).

Additionally, results were limited to Portuguese interactions. The choice to conduct this study exclusively in Portuguese was made to accurately reflect typical user interactions in Brazil, where large language models are frequently accessed in the native language. A previous study observed that while accuracy appears similar in Portuguese (63.1%) and English (60.2%), interaction patterns differed noticeably between languages (53). This observation underscores the need for further multilingual research to evaluate chatbot performance across diverse cultural and linguistic contexts.

Lastly, although this study performed 18,000 interactions over 30 consecutive days, accuracy estimates were ultimately bounded by the 60 unique items included in the benchmark. To address this limitation, future iterations of the benchmark will include a larger and more varied selection of items. This expansion aims to improve the breadth of content coverage while still allowing for meaningful longitudinal comparisons (30, 31). Nonetheless, the *post hoc* power analysis conducted for our study indicated that the current sample size was sufficient to achieve greater than 80% statistical power for detecting significant differences within the GLMM framework. This finding supports the robustness of the study's conclusions despite the limited number of benchmark items.

On the other hand, this study is the first to evaluate the performance of new LLMs, such as DeepSeek and MetaAI, in the comprehensive context of dental trauma. The 30-day longitudinal evaluation captured algorithmic variations and updates and captures temporal stability, a dimension absent from prior single-shot evaluations. This study also pioneered the assessment of calibration prompt influence on LLM accuracy and consistency in dental trauma. Finally, robust statistical analysis, using multiple performance metrics and generalized linear mixed models, provided a thorough understanding of the findings.

Clinically, the tested chatbots show promise as supplementary sources for dental trauma. They may be especially useful in urgent situations for professionals in public health networks who require immediate guidance, particularly in areas with limited specialist access, by providing rapid recommendations based on international guidelines such as IADT. They also have potential in educational settings, assisting parents, teachers, and students in adopting appropriate initial measures (21). Future applications will require integration with specialized databases and prospective validation. A multimodal system combining natural language processing and image analysis could overcome current accuracy limitations.

The rapid advancement and continual refinement of LLMs necessitate careful consideration of complex ethical issues, particularly concerning data privacy and data provenance in healthcare environments (54). It is essential for clinicians, patients, and developers to remain vigilant regarding the rights associated with personal data. These include the right to data ownership, the right not to be subjected to decisions based solely on automated processing, and the right to restrict the processing of their information. To address these challenges, governments and scientific societies must develop effective strategies that promote robust data protection measures. At the same time, these measures should facilitate the safe and ethical integration of LLMs into medical practice, ensuring that advancements in AI support rather than compromise patient rights and data security.

## 5 Conclusion

Microsoft Copilot and DeepSeek-V3 showed the highest accuracy and consistency over 30 days. Calibration prompts did not significantly increase accuracy or consistency of any model. Free LLMs currently available have potential as complementary tools for disseminating dental trauma information, provided their use is guided by reliable scientific sources and professional supervision.

## Data Availability

The original contributions presented in the study are included in the article/Supplementary Material, further inquiries can be directed to the corresponding author.
