# Supplementary material for: Performance of five free large language models in dental trauma: a 30-day longitudinal benchmark study
Source: Front Oral Health. 2025 Dec 15;6:1737114. doi: 10.3389/froh.2025.1737114 (PMC12745380; doi:10.3389/froh.2025.1737114)
Supplement: Supplementary file 1 [file Table1.docx]

Supplementary Material

**Supplementary Table 2:** Fillable TRIPOD-LLM checklist

| **Section** 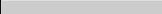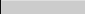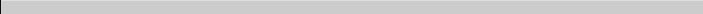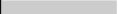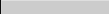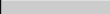 | **Item** | **Checklist Item** | **Research Design** | **LLM**  **Task** | **Page** |
| --- | --- | --- | --- | --- | --- |
| Title | | | | |  |
| Title | 1 | Identify the study as developing, fine-tuning, and/or evaluating the performance of an LLM, specifying the task, the target population, and the outcome to be predicted. | All | All | 1 |
| Abstract |  |  |  |  |  |
| Abstract | 2 | See TRIPOD-LLM for Abstracts | All | All | 1 |
| Introduction |  |  |  |  |  |
| Background | 3a | Explain the healthcare context / use case (e.g., administrative, diagnostic, therapeutic, clinical workflow) and rationale for developing or evaluating the LLM, including references to existing approaches and models. | All | All | 2 |
|  | 3b | Describe the target population and the intended use of the LLM in the context of the care pathway, including its intended users in current gold standard practices (e.g., healthcare professionals, patients, public, or administrators). | E H | All | 2 |
| Objectives | 4 | Specify the study objectives, including whether the study describes the initial development, fine-tuning, or validation of an LLM (or multiple stages). | All | All | 3 |
| Methods | | | | |  |
| Data | 5a | Describe the sources of data separately for the training, tuning, and/or evaluation datasets and the rationale for using these data (e.g., web corpora, clinical research/trial data, EHR data, or unknown). | All | All | 3 – Supplementary Table 2 |
|  | 5b | Describe the relevant data points and provide a quantitative and qualitative description of their distribution and other relevant descriptors of the dataset (e.g., source, languages, countries of origin) | All | All | 4 |
|  | 5c | Specifically state the date of the oldest and newest item of text used in the development process (training, fine-tuning, reward modeling) and in the evaluation datasets. | All | All | 4 |
|  | 5d | Describe any data pre-processing and quality checking, including whether this was similar across text corpora, institutions, and relevant socio-demographic groups. | All | All | NA |
|  | 5e | Describe how missing and imbalanced data were handled and provide reasons for omitting any data. | All | All | NA |
| Analytical Methods | 6a | Report the LLM name, version, and last date of training. | All | All | Table 1 |
|  | 6b | Report details of LLM development process, such as LLM architecture, training, fine-tuning procedures, and alignment | M D | All | Table 1 |

|  |  | strategy (e.g., reinforcement learning, direct preference optimization, etc.) and alignment goals (e.g., helpfulness, honesty, harmlessness, etc.). |  |  |  |
| --- | --- | --- | --- | --- | --- |
|  | 6c | Report details of how text was generated using the LLM, including any prompt engineering (including consistency of outputs), and inference settings (e.g., seed, temperature, max token length, penalties), as relevant. | M D E | All | 4 |
|  | 6d | Specify the initial and post-processed output of the LLM (e.g., probabilities, classification, unstructured text). | All | All | 4 |
|  | 6e | Provide details and rationale for any classification and, if applicable, how the probabilities were determined and thresholds identified. | All | C OF | NA |
| LLM Output | 7a | Include metrics that capture the quality of generative outputs, such as consistency, relevance, and accuracy, compared to gold standards. | All | QA IR DG SS MT | 4-5 |
|  | 7b | Report the outcome metrics' relevance to downstream task at deployment time and, where applicable, correlation of metric to human evaluation of the text for the intended use. | E H | All | 4-5 |
|  | 7c | Clearly define the outcome, how the LLM predictions were calculated (e.g., formula, code, object, API), the date of inference for closed-source LLMs, and evaluation metrics. | E H | All | 4-5 |
|  | 7d | If outcome assessment requires subjective interpretation, describe the qualifications of the assessors, any instructions provided, relevant information on demographics of the assessors, and inter-assessor agreement. | All | All | NA |
|  | 7e | Specify how performance was compared to other LLMs, humans, and other benchmarks or standards. | All | All | 4-5 |
| Annotation | 8a | If annotation was done, report how text was labeled, including providing specific annotation guidelines with examples. | All | All | 3 |
|  | 8b | If annotation was done, report how many annotators labeled the dataset(s), including the proportion of data in each dataset that were annotated by more than 1 annotator, and the inter- annotator agreement. | All | All | 3 |
|  | 8c | If annotation was done, provide information on the background and experience of the annotators or characteristics of any models involved in labelling. | All | All | 3 |
| Prompting | 9a | If research involved prompting LLMs, provide details on the processes used during prompt design, curation, and selection. | All | All | 4 |
|  | 9b | If research involved prompting LLMs, report what data were used to develop the prompts. | All | All | 4 |
| Summarizat ion | 10 | Describe any preprocessing of the data before summarization. | All | SS | 4 |
| Instruction tuning/Alig | 11 | If instruction tuning/alignment strategies were used, what were the instructions, data, and interface used for evaluation, | M D | All | NA |

| nment |  | and what were the characteristics of the populations doing evaluation? |  |  |  |
| --- | --- | --- | --- | --- | --- |
| Compute | 12 | Report compute, or proxies thereof (e.g., time on what and how many machines, cost on what and how many machines, inference time, floating-point operations per second (FLOPs)), required to carry out methods. | M D E | All | NA |
| Ethical Approval | 13 | Name the institutional research board or ethics committee that approved the study and describe the participant-informed consent or the ethics committee waiver of informed consent. | All | All | NA |
| Open Science | 14a | Give the source of funding and the role of the funders for the present study. | All | All | 9 |
|  | 14b | Declare any conflicts of interest and financial disclosures for all authors. | All | All | 9 |
|  | 14c | Indicate where the study protocol can be accessed or state that a protocol was not prepared. | H | All | N |
|  | 14d | Provide registration information for the study, including register name and registration number, or state that the study was not registered. | H | All | NA |
|  | 14e | Provide details of the availability of the study data. | All | All | 9 |
|  | 14f | Provide details of the availability of the code to reproduce the study results. | All | All | NA |
| Public Involvemen t | 15 | Provide details of any patient and public involvement during the design, conduct, reporting, interpretation, or dissemination of the study or state no involvement. | H | All | NA |
| Results | | | | |  |
| Participants | 16a | When using patient/EHR data, describe the flow of text/EHR/patient data through the study, including the number of documents/questions/participants with and without the outcome/label and follow-up time as applicable. | E H | All | 5-6 |
|  | 16b | When using patient/EHR data, report the characteristics overall and, for each data source or setting, and for development/evaluation splits, including the key dates, key characteristics, and sample size. | E H | All | 5-6 |
|  | 16c | For LLM evaluation that include clinical outcomes, show a comparison of the distribution of important clinical variables that may be associated with the outcome between development and evaluation data, if available. | E H | All | 5-6 |
|  | 16d | When using patient/EHR data, specify the number of participants and outcome events in each analysis (e.g., for LLM development, hyperparameter tuning, LLM evaluation). | E H | All | NA |
| Performanc e | 17 | Report LLM performance according to pre-specified metrics (see item 7a) and/or human evaluation (see item 7d). | All | All | 5-6 |
| LLM  Updating | 18 | If applicable, report the results from any LLM updating, including the updated LLM and subsequent performance. | All | All | NA |

| Discussion | | | | |  |
| --- | --- | --- | --- | --- | --- |
| Interpretatio n | 19a | Give an overall interpretation of the main results, including issues of fairness in the context of the objectives and previous studies. | All | All | 6-8 |
| Limitations | 19b | Discuss any limitations of the study and their effects on any biases, statistical uncertainty, and generalizability. | All | All | 6-8 |
| Usability of the LLM in context | 19c | Describe any known challenges in using data for the specified task and domain context with reference to representation, missingness, harmonization, and bias. | E H | All | 6-8 |
|  | 19d | Define the intended use for the implementation under evaluation, including the intended input, end-user, level of autonomy/human oversight. | E H | All | 6-8 |
|  | 19e | If applicable, describe how poor quality or unavailable input data should be assessed and handled when implementing the LLM, i.e., what is the usability of the LLM in the context of current clinical care. | E H | All | NA |
|  | 19f | If applicable, specify whether users will be required to interact in the handling of the input data or use of the LLM, and what level of expertise is required of users. | E H | All | NA |
|  | 19g | Discuss any next steps for future research, with a specific view to applicability and generalizability of the LLM. | All | All | 6-8 |

LLM = large language model; M = LLM methods; D = *de novo* LLM development; E = LLM evaluation; H = LLM evaluation in healthcare settings; C = classification; OF = outcome forecasting; QA = long-form question-answering; IR = information retrieval; DG = document generation; SS = summarization and simplification; MT = machine translation; EHR = electronic health record.

Note: For studies using existing LLMs, users should include reference(s) to reportable information if provided by the original developers or state that this information is not available.

**Supplementary Table 2.** Benchmark questions.

| 1. In cases of lateral luxation with severe displacement, should the primary tooth be extracted? 2. Can teeth in infraocclusion after replantation be subjected to orthodontic movement? 3. Does dental avulsion occur when the tooth is completely displaced from its socket? 4. Should primary teeth always be replanted after avulsion? 5. In cases of crown fracture without pulp exposure, should the tooth be restored as soon as possible? 6. Is external root resorption a common complication after dental trauma? 7. Is immediate extraction the main treatment for intrusive luxation? 8. Does the emergency treatment of a tooth with lateral luxation include surgical repositioning and rigid splinting? 9. Is milk an adequate medium for transporting an avulsed tooth to the dentist? 10. Do dental traumas always lead to pulp necrosis? 11. Do horizontal root fractures in the cervical third generally have a worse prognosis than root fractures in the middle and apical thirds? 12. In the case of avulsion of a permanent tooth with an open apex, is pulp canal obliteration on follow-up radiographs a positive outcome, indicating pulp healing? 13. Can trauma to primary teeth affect the underlying permanent teeth? 14. Is autotransplantation the best treatment option for tooth loss due to avulsion in adult patients? 15. Should dental splinting after extrusive luxation be maintained for at least 2 weeks? 16. Do teeth with extrusive luxation have a better pulp prognosis than teeth with intrusive luxation? 17. Should replantation of avulsed teeth ideally be performed within the first 30 minutes after trauma? 18. Is pulpotomy indicated in all cases of crown fracture with pulp exposure? 19. Are maxillary lateral incisors the most affected teeth in cases of avulsion? 20. Does a tooth with a vertical root fracture generally need to be extracted? 21. Is decoronation the first-choice treatment for all replanted avulsed teeth in infraocclusion? 22. Is root canal treatment the only option for complicated crown fractures in teeth with complete root development? 23. Can tooth discoloration after trauma indicate pulp necrosis? 24. Is intrusion the displacement of the tooth into the alveolus? 25. Does immediate treatment of dental trauma not affect the tooth’s prognosis? 26. Can dental fractures involve only the enamel? 27. Is ankylosis a commonly identified sequela in cases of subluxation and extrusive luxation? 28. Can dental trauma lead to external root resorption? 29. Does the absence of pain after dental trauma mean that no significant injury occurred? 30. Should endodontic treatment be performed 5 days after replantation of an avulsed tooth? 31. Do teeth with crown fractures without pulp exposure have a better prognosis than those with complicated crown fractures? 32. Does the use of a mouthguard reduce the risk of dental trauma in sports? 33. Are periapical lesions signs of pulp involvement after trauma? 34. Should flexible splints be removed after 2 weeks in cases of replantation? 35. Is antibiotic therapy necessary only in cases of avulsion, regardless of the presence of soft tissue trauma? 36. Are radiographs essential to assess the extent of dental trauma? 37. Does a crown-root fracture affect both the crown and the root of the tooth? 38. Is dental intrusion in primary teeth not in need of treatment? 39. Is monitoring of traumatized teeth necessary to identify late complications up to the first year post-trauma? 40. Should teeth with incomplete root development and compromised pulp vitality always undergo apexification? 41. Does dental trauma not affect the pulp vitality of the tooth? 42. Is decoronation indicated to treat teeth that suffered trauma and evolved to replacement resorption followed by ankylosis? 43. Does the absence of response to thermal tests after trauma indicate that the tooth’s nerve is intact? 44. Can dental trauma in permanent teeth require endodontic treatment? 45. Can pulp necrosis occur months after dental trauma? 46. Should the pulp vitality of young permanent teeth not be monitored regularly after trauma episodes? 47. Are repeated episodes of trauma rare and more common in adults? 48. Are Doppler ultrasound and laser flowmetry well-established and easily accessible technologies for pulp vitality monitoring? 49. Does an immature permanent tooth have considerable repair capacity after traumatic pulp exposure? 50. Is internal resorption a possible consequence of dental trauma? 51. Is the combination of two different types of traumatic injury affecting the same tooth more harmful than a single injury? 52. Is extraction of primary teeth due to trauma inevitable in all cases of severe luxation? 53. Should cone-beam computed tomography always be performed in cases of lateral luxation? 54. Is long-term monitoring of traumatized teeth unnecessary if the initial treatment was successful? 55. Is the use of systemic antibiotics after avulsion and replantation recommended to prevent infection-related reactions and reduce the occurrence of inflammatory root resorption? 56. Do crown fractures not modify the risk of pulp necrosis and infection in teeth with concussion or subluxation and complete root formation? 57. Is the use of clinical photographs highly recommended for initial documentation of the traumatic injury and follow-up examinations? 58. Will the use of positioning devices make no difference during radiographic evaluations of a traumatized tooth? 59. Can traumatized teeth present color changes over time? 60. Does immediate treatment after dental trauma increase the chances of successful recovery? |
| --- |

**Supplementary Table 3.** Post hoc power analysis.

|  | Estimate | Std. Error | z value | Pr(>\|z\|) | detectavel_80 | detectavel_or |
| --- | --- | --- | --- | --- | --- | --- |
| (Intercept) | 5.98424246 | 0.8672093 | 6.9005745 | 0.000000000005179263 | 2.4281861 | 11.338296 |
| ModeloCopilot | 0.96971365 | 0.1771730 | 5.4732596 | 0.000000044183204686 | 0.4960843 | 1.642278 |
| ModeloDeepseek | 0.62900388 | 0.1702953 | 3.6936070 | 0.000221095431793934 | 0.4768268 | 1.610954 |
| ModeloGemini | -0.38732214 | 0.1559741 | -2.4832467 | 0.013019088083580392 | 0.4367274 | 1.547634 |
| ModeloMeta | 0.13150274 | 0.1621239 | 0.8111249 | 0.417293940225414717 | 0.4539470 | 1.574514 |
| PromptCom | -0.13842721 | 0.1586249 | -0.8726703 | 0.382842826199666475 | 0.4441496 | 1.559164 |
| ModeloCopilot:PromptCom | -0.05426463 | 0.2453849 | -0.2211408 | 0.824982769977470554 | 0.6870778 | 1.987898 |
| ModeloDeepseek:PromptCom | 0.10660021 | 0.2386946 | 0.4465966 | 0.655166350896573046 | 0.6683450 | 1.951006 |
| ModeloGemini:PromptCom | 0.12701144 | 0.2190248 | 0.5798951 | 0.561985337032797627 | 0.6132695 | 1.846459 |
| ModeloMeta:PromptCom | 0.13842353 | 0.2280353 | 0.6070267 | 0.543833188587486527 | 0.6384989 | 1.893636 |
